# Transcriptomic Modification in the Cerebral Cortex following Noninvasive Brain Stimulation: RNA-Sequencing Approach

**DOI:** 10.1155/2016/5942980

**Published:** 2016-12-29

**Authors:** Ben Holmes, Seung Ho Jung, Jing Lu, Jessica A. Wagner, Liudmilla Rubbi, Matteo Pellegrini, Ryan Jankord

**Affiliations:** ^1^Applied Neuroscience, Warfighter Interface Division, 711th Human Performance Wing, Air Force Research Laboratory, Wright-Patterson AFB, Dayton, OH 45433, USA; ^2^Department of Molecular, Cell, and Developmental Biology, University of California, Los Angeles, CA 90095, USA

## Abstract

Transcranial direct current stimulation (tDCS) has been shown to modulate neuroplasticity. Beneficial effects are observed in patients with psychiatric disorders and enhancement of brain performance in healthy individuals has been observed following tDCS. However, few studies have attempted to elucidate the underlying molecular mechanisms of tDCS in the brain. This study was conducted to assess the impact of tDCS on gene expression within the rat cerebral cortex. Anodal tDCS was applied at 3 different intensities followed by RNA-sequencing and analysis. In each current intensity, approximately 1,000 genes demonstrated statistically significant differences compared to the sham group. A variety of functional pathways, biological processes, and molecular categories were found to be modified by tDCS. The impact of tDCS on gene expression was dependent on current intensity. Results show that inflammatory pathways, antidepressant-related pathways (GTP signaling, calcium ion binding, and transmembrane/signal peptide pathways), and receptor signaling pathways (serotonergic, adrenergic, GABAergic, dopaminergic, and glutamate) were most affected. Of the gene expression profiles induced by tDCS, some changes were observed across multiple current intensities while other changes were unique to a single stimulation intensity. This study demonstrates that tDCS can modify the expression profile of various genes in the cerebral cortex and that these tDCS-induced alterations are dependent on the current intensity applied.

## 1. Introduction

The number of publications about therapeutic and beneficial effects of transcranial direct current stimulation (tDCS) on the central nervous system has dramatically increased over the past several years. One of the main reasons for the attractiveness of tDCS as a tool to modify neuroplasticity and neuronal activity [1] is that tDCS is a noninvasive brain stimulation technique that is well tolerated and easily employed with other peripheral therapies [2, 3]. Studies have highlighted tDCS as an alternative treatment method for schizophrenia [4], Alzheimer's [5], major depressive disorder [6, 7], stroke [8], and other neurological disorders. Moreover, tDCS has shown potential for aiding cognitive performance in healthy individuals with previous studies showing improvement of sustained attention [9], enhanced working memory [10], and enhanced functional connectivity in spatial navigation networks [11].

Animal-based studies have been conducted to identify underlying electrophysiological mechanisms by which tDCS produces its beneficial effects on brain performance. Using rodent models, studies have shown that tDCS increased cortical excitability [12], modulated motor-evoked potentials [13], stimulated synaptic mechanisms [11], and enhanced hippocampal synaptic plasticity [14]. These results suggest that tDCS may be a potential therapeutic option for patients with neurological and neuropsychiatric diseases and disorders. Of interest, effects of tDCS were still observed following the cessation of stimulation and persisted for hours after tDCS treatments [14–16].

Compared to the electrophysiological mechanisms, less is known about the molecular and cellular pathways affected by tDCS. Spezia Adachi et al. [17] reported that hippocampal TNF-*α* levels were decreased by repeated stimulation (20 min/day for 8 days). Another rodent-based study showed that tDCS reduced middle cerebral artery occlusion-induced expression of hemichannel pannexin-1 in hippocampal neurons in addition to preventing the occlusion-induced decrease in dendritic spine density [18]. Another occlusion study reported that tDCS affected MAP-2 and GAP-43 expression in some brain regions of cerebral ischemic rats [19]. Overall, very few studies have been conducted to analyze tDCS-induced changes in protein and gene expression. Therefore, the purpose of this study was to assess the impact of anodal tDCS on whole transcriptomic profiles within the cerebral cortex, on which an electrode was attached, and provide insight into the molecular mechanisms of anodal tDCS at various current intensities.

For this study, anodal tDCS was applied at several different intensities (sham, 250 *μ*A, 500 *μ*A, and 2,000 *μ*A). Following stimulation, dissects of the cerebral cortex were collected, RNA was extracted, and RNA-Seq analysis was completed using next-generation sequencing (NGS) technology, obtained with the Illumina RNA-sequencing system. Data from the sequencer was analyzed with the Tuxedo suite tools to produce gene counts and fold changes, which were analyzed using multiple bioinformatics databases. Results show that different intensities of tDCS can modulate a massive number of genes representing a collection of functional biological pathways.

## 2. Material and Methods

### 2.1. Animals

Male Sprague Dawley rats (9-10 weeks old) were obtained from Charles River Laboratories (Wilmington, MA) and housed in the Wright-Patterson Air Force Base (WPAFB) animal facility. Rats between 300 and 500 g were used for these experiments, doubly housed with ad libitum access to food and water, and maintained on a 12 : 12-hour light-dark cycle. Rodents were randomly assigned to Sham, 250 *μ*A, 500 *μ*A, and 2,000 *μ*A tDCS groups (*n* = 7-8 per group), and all experiments were performed during the light cycle and done by 12 PM. All procedures were approved by the WPAFB Institutional Animal Care and Use Committee and performed in accordance with the National Institute of Health standards and the Guide for the Care and Use of Laboratory Animals (National Research Council, 2013).

### 2.2. Electrode Implantation Surgery and Transcranial Direct Current Stimulation

Rodents were anesthetized with isoflurane (Piramal, Shope Med Vet, Mettawa, IL) at an average of 2-3% and maintained during the stimulation. A head incision was made to expose the implantation area and a head electrode (approximately 5 × 5 mm, Axelgaard Manufacturing Factory Ltd., Fallbrook, CA) was placed with the center on the sagittal suture, 2.5 mm caudal bregma. The head electrode was cemented to the skull using Metabond Adhesive Luting Cement (Parkell Inc., Edgewood, NY). Once the cement was dry, acrylic (Stoelting Co. Fisher Scientific, Pittsburgh, PA) was added over the cement to create a head cap. A head clamp (AFRL, WPAFB, OH) was attached to the skull in order to anchor the acrylic and maintain the integrity of the head cap.

### 2.3. Whole Transcriptome RNA-Sequencing Performance

After anodal tDCS for 20 min was completed, rats were immediately euthanized and a portion of the cerebral cortex under the anodal tDCS location was collected and frozen on dry ice. Total RNA was extracted from the cerebral cortex utilizing the RNeasy Mini Kit, following the manufacturer's protocol (Qiagen, Valencia, CA). RNA samples from two animals were combined together for RNA-Seq analysis (*n* = 4 per group) to allow for sufficient volume of total RNA and, then, overnight shipped to the Next Generation Sequencing (NGS) facility at the UCLA, CA. At the NGS facility, the library was constructed, and samples were multiplexed and tagged with standard Illumina tags. Samples were sequenced using Illumina next-generation RNA-sequencing.

### 2.4. Bioinformatic Analysis

Data was first obtained in  .qseq file format from the RNA sequencer, demultiplexed, and stored in  .fastq file format. Tophat (version 2.0.6) [20] together with bowtie (version 0.12.8) [21] was used to align reads to UCSC genome browser (rat genome assembly RGSC5.0/rn5). The sequencing resulted in about 54 million raw reads for each sample (mean ± SED: 54,905,147 ± 1,204,160, *p* > 0.05) and more than 40 million of uniquely mapped reads (mean ± SED: 44,790,986 ± 930,596, *p* > 0.1). Only uniquely mapped gene reads (mean ± SED: 82%  ± 0.25%) were kept. The gene reads were quantified by HTSeq (version 0.5.3p9) [22] with Ensembl 75 gene sets. Differential gene expression analysis was performed using DESeq2 (ver. 1.4.5) [23] and genes that had no gene reads across all samples were discarded. As previously recommended [23], genes with more than 1.2 log_2_ fold change in expression and an adjusted *p* value of less than 0.1 were classified as significantly differentially expressed.

DAVID Bioinformatics [24] and PANTHER Classification System [25] were used to analyze differentially expressed genes. These databases were used to classify the genes into functional groups, providing information about their biological function. When using DAVID, the enrichment score cutoff was set to be 1.3 (which corresponds to a corrected *p* value of 0.05).

The PANTHER Classification System (http://pantherdb.org/) was also used to classify differentially expressed genes [26]. Lists of gene names were entered and analyzed with the organism selection of* Rattus norvegicus*. The PANTHER database recognized 418, 252, and 450 genes, respectively, from the gene lists from 250, 500, and 2,000 *μ*A samples (at least 77% recognition rate). Genes recognized by the PANTHER were classified according to pathway, biological process, and molecular function. The PANTHER database contains about 12,000 protein families, which are also divided into more than 83,000 functionally distinct protein subfamilies [25, 27].

### 2.5. Weighted Gene Coexpression Network Analyses (WGCNA)

Normalized expression values for genes from DESeq data were used to construct signed coexpression networks using the WGCNA package in R [28]. Low expression genes were first excluded from the analyses to remove noise (the number of total genes decreased from 17,749 to 12,714). Network construction and module detection were done based on the WGCNA package manuals. Briefly, after the 1st step of data input and cleaning was completed, the step-by-step construction of the gene network and identification of modules was used to construct a weighted gene network with a soft-thresholding power of 5, for which scale-free topology fit index (SFT. *R* ^2^) was 0.9250. A dissimilarity calculated based on the topological overlap matrix transformed from the adjacency was used for hierarchical clustering to produce a hierarchical clustering tree of genes that was then used to identify the modules with a set of the minimum module size as 30. The Dynamic Tree Cut was used to identify similar modules; their eigengenes were calculated, clustered based on their correlation, and, then, merged into modules if their correlation was greater than 0.75. This reduced the module number from 40 dynamic modules to 15 merged modules. An association of individual genes in each module with our trait, tDCS current intensity, was quantified by defining gene significance as the absolute value of the correlation between the gene and the trait (see Figure 6(a)). A correlation of the module eigengene and the gene expression profile was calculated and defined as a quantitative measure of module membership. Modules that had a high significance for tDCS current intensity with genes as well as high module membership were identified and 2 modules with the top 2 highest correlations (*r* ≤ 0.7 with *p* ≤ 0.005) were used to perform gene ontology (GO) enrichment analysis (Table 4). The top 30 genes from the highest module were visualized with VisANT 5.0 [29] for gene connections (threshold of 0.35).

### 2.6. Statistical Methods

Results were analyzed both from HTSeq-count's list of names, *p* values, and fold changes and from the pathways indicated by DAVID and PANTHER. The data were globally normalized before being run through Tophat. Once Tophat and HTSeq-count were run on the RNA-Seq data, log_2_ fold changes (log_2_⁡FC) and *p* values were determined to gauge the difference between sham and experimental groups. Only those genes which fulfilled the minimum requirements for significance (see Bioinformatic Analysis for details) were considered for analysis.

## 3. Results

**Figure 1:**
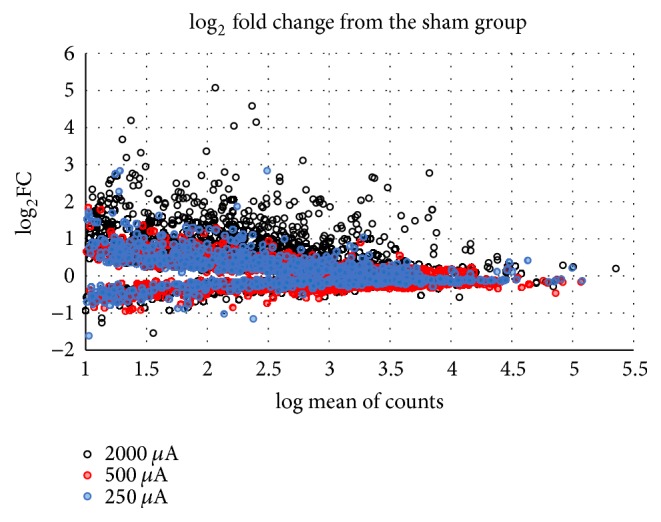
Comparison of genes altered by various tDCS intensities. For this plot, DESeq countable data was used to calculate log_10_ mean of counts and log_2_ fold change. The *x*-axis is log_10_ mean of counts and the *y*-axis is log_2_ fold change from sham group. For this plot, the genes with an expression level ≥ 10 counts and *p* value (one-tailed *t*-test between each tDCS and sham group) less than 0.05 were selected to draw the plot. The greater distance from 0 log_2_⁡FC represents a larger change in gene expression levels from sham.

When log_2_⁡FC were compared between sham animals and those stimulated at 3 different currents, 947, 945, and 948 genes were identified from the groups of 250 *μ*A, 500 *μ*A, and 2000 *μ*A, respectively. When the data were plotted (Figure 1), there was a clear separation between those stimulated at 250 or 500 *μ*A, and those stimulated at 2000 *μ*A. The stimulation at 2000 *μ*A shows a marked overall increase in fold change. On the other hand, genes induced by the 250 and 500 *μ*A intensities of anodal tDCS were expressed more when stimulated by the 2000 *μ*A intensity of anodal tDCS.

### 3.1. Pathway Analysis of DAVID Bioinformatics

**Figure 2:**
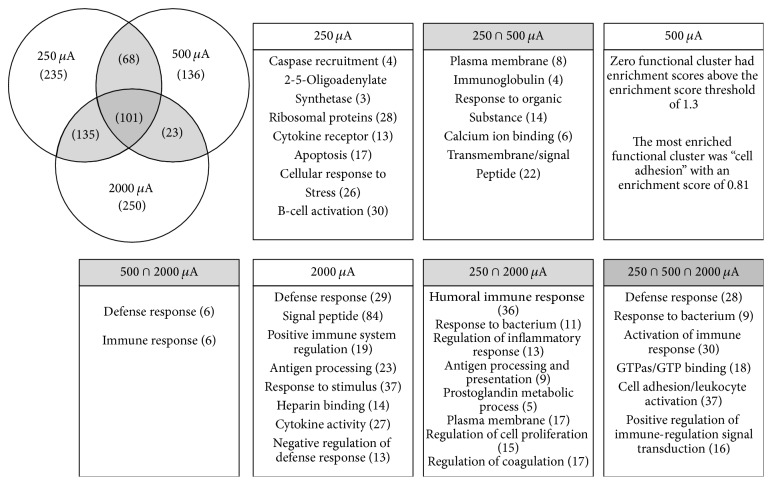
Highly enriched terms from DAVID's Bioinformatics functional clustering with total number of genes listed in parentheses. Listed in parentheses is the number of genes induced by each current stimulation only (250 *μ*A only, 500 *μ*A only, and 2000 *μ*A only).

DAVID Bioinformatics analysis was used to classify significantly expressed genes into functional clusters [24] as shown in Figure 2. Overall, each tDCS group included unique genes and, thus, unique functional clusters, which were not expressed in any other group. It is also true that some genes were detected in more than one group. Groups with a large number of associated genes include transmembrane/signal peptide (22 genes) in the intersection of 250 and 500 but not 2000 *μ*A, humoral immune response (36 genes) in the intersection of 250 and 2000 but not 500 *μ*A, defense and immune response (each with 6 genes) in the intersection of 500 and 2000 but not 250 *μ*A, and cell adhesion/leukocyte activation (37 genes) in the intersection of all 250, 500, and 2000 *μ*A. A number of clusters were unique to each tDCS current intensity: notably ribosomal proteins with 28 genes in the 250 *μ*A group, signal peptides with 84 genes in the 2000 *μ*A group, and, while not as enriched as the other clusters, cell adhesion in the 500 *μ*A group. Interestingly, no functional cluster was detected from the group 500 *μ*A only with significant enrichment.

### 3.2. Pathway Analysis of PANTHER Classification

**Figure 3:**
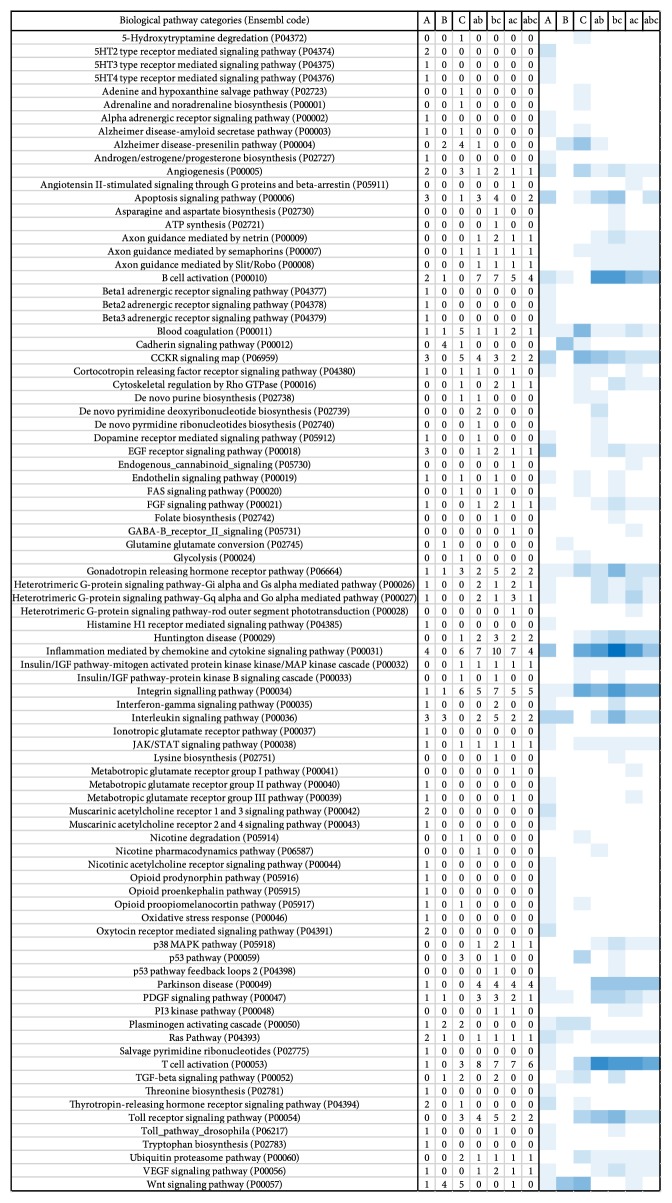
Heatmap from PANTHER biological pathway analysis. Heatmap was made with Plotly (available online at https://www.plot.ly/) with a white-to-blue color theme and drawn from RNA-Seq results from each group of 250 *μ*A (A) only, 500 *μ*A (B) only, and 2000 *μ*A (C) only in addition to the intersections of 250 & 500 *μ*A (ab), 250 & 2000 *μ*A (ac), 500 & 2000 *μ*A (bc), and all 3 current intensities (abc).

The PANTHER database was used to perform functional pathway analysis on the selected differentially expressed genes, and the data was consolidated into a heatmap (Figure 3). Several similarities are readily evident by viewing the heatmap; genes relating to inflammation mediated by the chemokine and cytokine pathway fell into the largest or second-largest functional pathways in some intersections and groups of 250 *μ*A and 2000 *μ*A. Another highly represented pathway in the intersection of 250 and 500 *μ*A, 250 and 2000 *μ*A, and all 250, 500, and 2000 *μ*A was T-cell activation, where it was the largest or second-largest functional pathway. Other large functional pathways included the interleukin signaling, CCKR signaling map, apoptosis signaling, and EGF-receptor signaling pathways in the 250 *μ*A group; cadherin signaling and Wnt signaling pathways in the 500 *μ*A group; and integrin signaling pathway in the 2000 *μ*A group and the intersection of 250, 500, and 2000 *μ*A. Additionally, a number of neurotransmitter functional pathways were affected by tDCS, such as serotonergic, adrenergic, and dopaminergic pathways.

**Figure 4:**
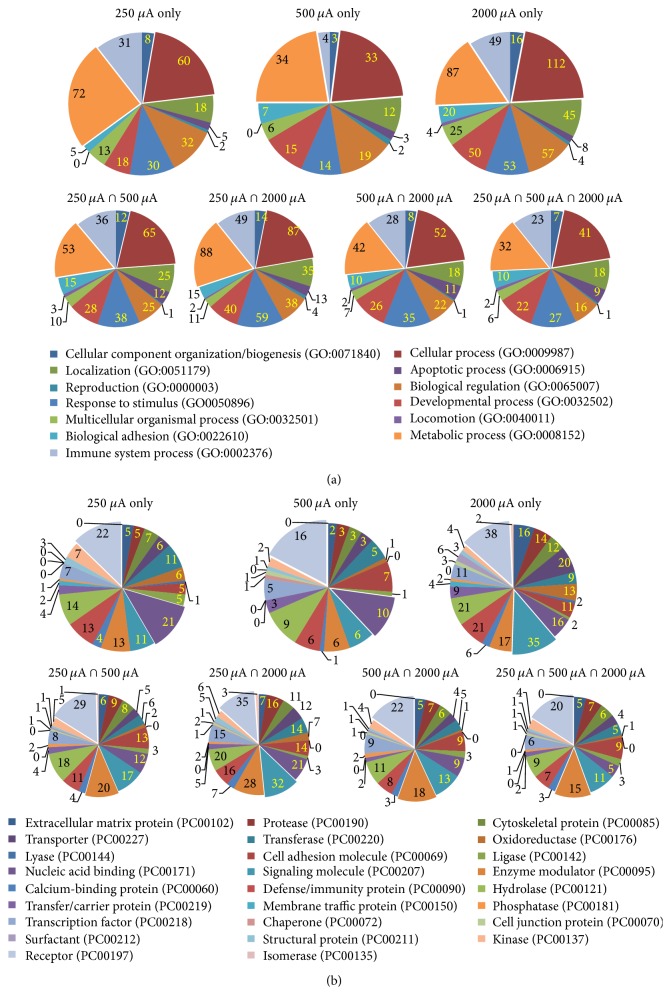
Results from PANTHER classification analyses. (a) Functional biological process categories. (b) Molecular functional protein categories.

PANTHERDB classification analysis was used to visualize functional biological processes (Figure 4(a)) and molecular protein categories (Figure 4(b)). In the functional biological process categories, similarities were detected between groups. In particular, for every intensity, the functional biological processes with the greatest number of associated genes were metabolic and cellular processes (Figure 4(a)). Immune system process, response to stimulus, developmental process, and localization were also categories showing a consistently large number of genes, with the exception of immune system processes in the 500 *μ*A group only.

**Figure 5:**
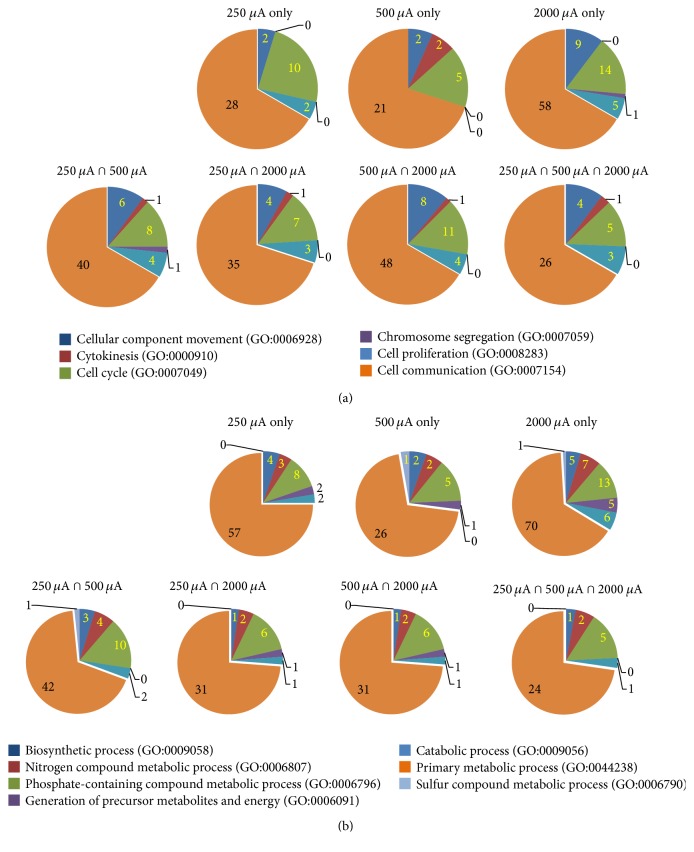
Two largest categories of gene ontology (GO) functional biological process analyses. (a) Six subcategories were identified from the cellular category. (b) Seven subcategories were identified from the metabolic process category.

In order to further explore the makeup of the largest functional biological process categories, genes associated with cellular (Figure 5(a)) and metabolic (Figure 5(b)) processes were listed and visualized with pie charts. Even within categories, similarities between groups are evident. In the cellular processes, cell communication dominates every group, with over half of the genes associated with it. Likewise, cell cycle and cellular component movement categories have relatively large numbers of genes in all groups. The genes associated with cellular communication, the largest category in every group, are listed in Table 1.

Metabolic process groups were dominated by one particular category. In particular, primary metabolic processes had more than half the genes in every group, often approaching three-quarters of all the genes. Aside from this huge group, phosphate-containing compound metabolic processes, nitrogen compound metabolic processes, and biosynthetic processes were identified from the groups of 250 *μ*A, 500 *μ*A, and 2000 *μ*A and the intersection of 250 and 500 *μ*A. These processes frequently have relatively large numbers of genes associated with them. Genes associated with primary metabolic processes (the largest subgroup) are listed in Table 2.

Consistent patterns can be found in the functional protein categories shown in Figure 4(b). In all groups, receptor proteins had the greatest number of associated genes. Beyond this group, there is significantly more variation between groups, but it is typical that phosphatase, defense/immunity protein, signaling molecule, and transfer/carrier proteins have a large number of genes associated with them. The genes associated with receptors, the largest category of molecular functional proteins, are listed in Table 3. The PANTHER analysis of our whole transcriptomic data showed that major subgroups within the receptor protein class were G-protein coupled receptors, cytokine receptors, ligand-gated ion channels, and protein kinase receptors.

### 3.3. Weighted Gene Coexpression Network Analyses (WGCNA)

**Figure 6:**
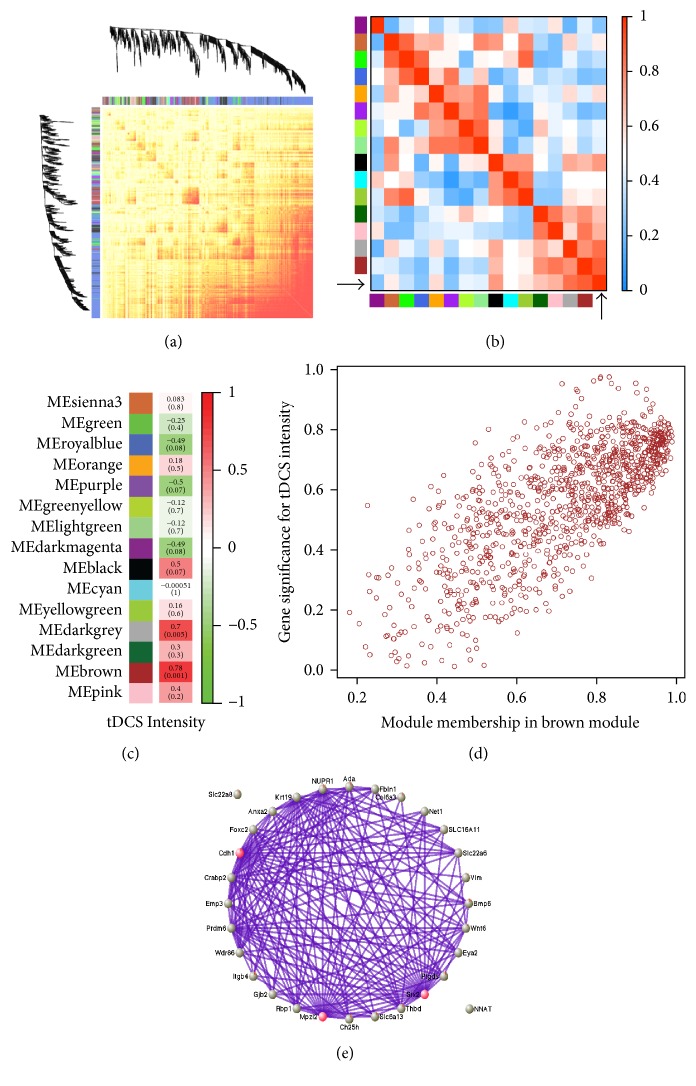
Weighted Gene Coexpression Network Analysis (WGCNA). (a) Network heatmap plot. Each branch in the hierarchical clustering dendrograms corresponds to each of 15 modules determined by the WGCNA. The color bars between the dendrograms and the heatmap plot represent the color-coded eigengene-based connectivity. Progressively more red color indicates highly interconnected genes in each module. (b) Heatmap plot of the adjacencies in the eigengene network including the trait tDCS current intensities. Each row and column in the heatmap indicate one module eigengene and the last row and column represent the trait tDCS current intensities. Negative and positive correlation are represented by green and red colors, respectively, as shown on the indicator (range: 0.0–1.0). (c) Module-trait (tDCS current intensity) relationship. Each row corresponds to a module eigengene, and each cell contains the corresponding correlation (top) and *p* value (bottom in parentheses) calculated by the WGCNA package. (d) A scatterplot of gene significance (GS) for tDCS intensity versus module membership (MM) in the brown module. A highly significant correlation between GS and MM in the brown module (correlation = 0.74; *p* value = 8.2*e* ^−186^) was detected. (e) The network connections among the most connected genes in the brown module, generated by the VisANT software (version 5.5). The plot shows network connections whose topological overlap is above the threshold of 0.35, and the red-colored genes are the top 3 genes that have the greater number of connections.

To evaluate relationships between gene expression and tDCS intensities, we conducted WGCNA analysis. A network heatmap plot (Figure 6(a)) was created by WGCNA showing overall module-related gene branches in hierarchical clustering dendrograms. WGCNA determined a total of 15 merged modules. An eigengene network heatmap (Figure 6(b)) showed the correlations among these 15 module eigengenes and tDCS intensities (indicated by black arrows). As shown in Figure 6(b), tDCS current intensity is significantly correlated with only the brown and dark grey module eigengenes.  Figure 6(c) shows their correlations and *p* values with the tDCS current intensity trait, and only 2 modules showed absolute correlation values equal to or greater than 0.7 with *p* values less than 0.01: the brown module (*r* = 0.78, *p* = 0.001) and dark grey module (*r* = 0.7, *p* = 0.005). Gene significance (an indicator for biological significance) for tDCS current intensity was highly and significantly correlated with module membership (an indicator for gene connectivity with the module eigengene) in the brown module (Figure 6(d)). Because the brown module showed the highest level of interconnectedness, the top 30 genes were pulled out from the brown module and exported for visual analyses (Figure 6(e)). Among these top 30 genes, the 3 most highly connected genes were six2, cdh1, and mpzl2 (red-colored balls).

Gene ontology (GO) annotation of the brown and dark grey modules was used to identify the functional pathways of genes in these modules (Table 4). The analysis determined that the top 3 most highly ranked pathways in the brown module were labeled: extracellular region, proteinaceous extracellular matrix, and extracellular space. In the dark grey module, the top 3 pathways were response to drug, eosinophil chemotaxis, and eosinophil migration. Among the top 20 ranked pathways in the combined brown and dark grey modules, the pathways extracellular region (299 genes), response to organic substance (245 genes), tissue development (178 genes), response to lipid (119 genes), and extracellular space (116 genes) were found to have a great number of genes.

## 4. Discussion

This study was conducted to investigate how anodal tDCS affects whole transcriptome expression in the cerebral cortex and to identify the functional biological pathways modulated by brain stimulation. Overall, a series of bioinformatics analyses of the RNA-Seq data demonstrated that tDCS resulted in significant transcriptomic modifications in the cortex and identified multiple biological pathways, such as various receptor signaling, metabolism, cytokine/chemokine signaling, cell adhesion, and transmembrane signaling. Additionally, our results also discovered that different current intensities have various effects on transcriptomic expression levels and, thereby, demonstrated that the patterns of gene induction and the magnitude of the response are dependent upon tDCS current intensity.

We first employed the DAVID pathways tool to identify functional clusters of genes and found 32 functional clusters that were affected by tDCS at the different intensities. However, not all functional clusters were significantly enriched across all intensities. Cluster analysis with DAVID Bioinformatics resulted in 25, 12, and 21 clusters from the groups of 250 *μ*A, 500 *μ*A, and 2000 *μ*A, respectively (Figure 2). All intensities stimulated, to some degree, inflammatory/immune pathways, such as the activation of immune response (30 genes), cell adhesion/leukocyte activation (37 genes), and positive regulation of immune-regulation signal transduction (16 genes). The stimulation at higher intensities affected a greater number of functional clusters related to inflammatory and immune responses (42%, 50%, and 57% of clusters were related in the 250, 500, and 2000 *μ*A groups, resp.). Additionally, the inflammatory and immune pathways were significantly more upregulated in the 500 and 2000 *μ*A groups. This result may be used to explain previous findings showing that higher intensities induced negative effects (e.g., tissue damage) on the rodent cortex [30]. Our histology data (unpublished) showed no visible lesion at or below 300 *μ*A, but visible lesion on the cortex was greater when the intensity was increased to 500 *μ*A.

A growing body of evidence suggests an antidepressant effect of tDCS in humans [7] and rodents [31], and our data may provide more evidence to support the antidepressant effect of tDCS. It was reported that potential therapeutic effects of tDCS on some psychiatric disorders, such as major depressive disorder, were induced through the regulation of GTP signaling pathways in the cerebral cortex [32]. The modulation of GTP signaling also requires the activation of some ion channels, especially calcium channels [33]. The results of the DAVID functional clustering indicated that tDCS at all intensities affected GTP signaling pathways. Moreover, our data showed that, in particular, 250 and 500 *μ*A intensities affected more signaling pathways related to calcium ion binding and transmembrane/signal peptide. Our results suggest that tDCS could modulate calcium channel-to-GTP signaling, providing a potential mechanism by which tDCS could provide therapeutic and beneficial effects on some psychiatric disorders. Additional studies should be warranted to clarify the interaction between tDCS, psychiatric disorder, and these pathways, based upon the findings reported here.

DAVID Bioinformatic analysis was also able to detect gene pathways relating to inflammation, and chemokine and cytokine signaling were differentially expressed following stimulation at different intensities. These pathways are important for metabolism and intercellular communication and can have effects on neuroplasticity, LTP, and the development of new neural pathways [12]. These pathways can serve both as indicators of the process by which neural plasticity might be affected and as warning signs of neural damage when establishing safe parameters for current intensity and duration of tDCS. Moreover, increased levels of neural inflammation, as shown at the higher intensities, have been reported to be related to cognitive impairment, reduced cognitive performance, and depression, thus inducing negative behavioral changes [34–37]. The upregulation of proinflammatory cytokines and pathways correlated with observed lesioning of the brain in our study but was also observable when no visible lesions occurred. These pathways can indicate that some damage is taking place, and the amount by which these pathways are upregulated can indicate how risky a current intensity might be for a subject.

We also analyzed our RNA-sequencing data by using PANTHER database in addition to DAVID Bioinformatics. The use of PANTHER database allowed for our data to be analyzed using additional databases and allowed results to be produced using slightly different bioinformatic methods for interpreting the RNA-sequencing data. The PANTHER database analysis revealed that tDCS modified about 90 different functional biological pathways, 13 biological processes, and 26 protein molecule categories in the cortex. As shown in Figure 4, the results showed similar patterns as seen in the DAVID analysis, such as inflammatory/immune signaling and intracellular signaling pathways. Many genes in the intersection of 250 and 500 *μ*A function as intracellular signaling, while most of the genes detected in the intersection of all intensities were classified as immune and inflammatory signaling. As discussed above, literature [30] and our unpublished data showed that tDCS at 2000 *μ*A induced some degree of damage on the cortex (especially in the upper layers). This, in addition to the data from DAVID functional analysis, suggests that some of the gene expression changes observed at 2000 *μ*A are the result of tissue damage, induced possibly by too high current intensity, and may be, thus, confounding effects of increased current intensity.

The PANTHER pathway analysis showed that tDCS at different intensities modified signaling pathways of some neurotransmitter receptors, including serotonergic, adrenergic, GABAergic, dopaminergic, acetylcholinergic, glutamate, and oxytocin receptors. These receptors, such as glutamate receptors, are required to form synaptic plasticity with the Ras signaling pathway, and our data discovered this signaling pathway is affected by anodal tDCS. Interestingly, the 250 *μ*A intensity showed the greatest expression of Ras signaling pathway-related genes, and as the current intensity was increased, fewer genes were expressed. These findings may suggest that neurotransmitter receptors related to synaptic plasticity are affected at all levels of stimulation but that signaling pathways related to synaptic plasticity are more dependent upon stimulation current intensity.

As identified by the PANTHER biological analysis tool, the two largest biological process categories modified by tDCS at all current intensity levels were cellular and metabolic processes (Figure 4(a)). When these categories were further broken into subcategories (Figure 5), we found that most genes in cellular and metabolic processes were subclassified into cell communication and primary metabolic process categories (at least more than 70% in each subcategory). The detected genes of these subcategories are listed on Tables 1 and 2. Briefly, genes related to intracellular signaling pathways, receptors, and inflammatory response were detected in the subcategory of cell communication and genes related to transmembrane proteins, metabolic pathways, and intracellular signaling pathways were detected in the subcategory of primary metabolic process. These results indicate that tDCS at all current intensity levels is effective to modify both cell metabolism and communication.

PANTHER molecular protein analysis also produced significant findings, as shown in Figure 4(b). The PANTHER analysis showed that 20 molecular classes in the cortex were affected by tDCS. The largest protein class designation was “receptor” across all the groups, followed by nucleic acid binding, signaling molecule, and enzyme modulator classes. We further analyzed the largest protein category, receptor, and found four subcategories: G-protein coupled receptor, cytokine receptor, ligand-gated ion channel, and protein kinase receptor. One of the largest subclasses within the receptor category, G-protein coupled receptor, is known to be related to neurotransmitter receptors, metabolic pathways, and extracellular and intracellular signaling pathways. An interesting finding from the receptor protein class was that one of the largest protein subclasses affected was cytokine receptors. This result, that is, tDCS significantly effects a variety of receptor pathways, may support the results obtained from PANTHER analyses showing significant changes in cell communication. Future studies should be warranted to elucidate underlying mechanisms of tDCS-related expression of these cytokine receptors and other receptors in the cortex.

To evaluate the effects of tDCS intensity on transcriptomic expression, WGCNAs were calculated and from them, 15 modules were identified. Genes from the top 2 modules had pathway analysis performed on them by GO annotation. As shown in Table 4, some of the functional pathways in given modules had already been detected as important pathways by DAVID and PANTHERDB. In particular, the functional pathways response to organic substance from the brown module and heparin binding from the dark grey module had been detected by the DAVID functional analysis (Figure 2). While some of the pathways detected by WGCNA were unique, for example, extracellular space, extracellular region, tissue development, vasculature development, and blood vessel development in the brown module, many other pathways have clear analogues from the other bioinformatics assays. The cytokine and chemokine signaling pathways were detected by all three methods we conducted for this study. These similarities between results obtained from WGCNA and those from DAVID bioinformatics and PANTHER database analyses highlight the result that tDCS induces change in signaling pathways, especially involving cytokine and chemokine receptors, which in turn have important effects on how neural cells are communicating, metabolizing, and structuring.

While several categories of genes, that is, those involved in metabolism or immune response, were significantly upregulated at all intensities, this does not indicate that modification of gene expression by tDCS is not dose-dependent. Rather, a single category of genes might be upregulated at all intensities, but the specific genes belonging to that category might be different from intensity to intensity. Indeed, some particular pathways can be upregulated at all current intensities yet have different outcomes depending on the presence or absence of regulatory genes, genes which are demonstrably dose-dependent. One notable example of a functional pathway activated in an intensity-dependent manner, discovered through DAVID analysis, is the NLRP3 inflammasome. Involved in neurogenesis, inflammation, and neural plasticity, the inflammasome leads to the production of IL-1*β*, which can further upregulate inflammatory pathways, polarizing macrophages to the aggressive M1 phenotype, recruiting other immune system cells, and leading to tissue damage. At 250 and 500 *μ*A, this pathway was regulated by IL-10 and DUSP1, and IL-1 *β* was not significantly upregulated from the sham group. At 2000 *μ*A, the picture is reversed: the regulatory elements were similar to the sham group, and IL-1 *β* showed 6-fold upregulation. Thus, the dose dependence is clearly shown to be a factor in the change of expression levels for some, but not all, of the genes significantly affected by tDCS. Modulation in treatment should therefore involve frequency stimulation, but only with acceptable parameters for current intensity, determined through experiment.

Moreover, our findings may provide evidence to support long-lasting effects of one-time anodal tDCS on brain performance, such as neuroplasticity. One recent electrophysiologic study [14] showed beneficial effects of one-time anodal tDCS on neuronal plasticity and long-term potentiation (LTP), and acute tDCS-induced changes were reported to last for hours [12, 14, 38]. This enduring effect of one-time brain stimulation may be induced by changes in genes, thus protein, expression, and pathways that play an important role in neuronal plasticity and LTP. Although more supporting evidence is warranted to be provided, the current study shows that even one-time anodal tDCS modified the expression of a variety of genes and pathways and thus will suggest that enduring effects of one-time anodal tDCS in the brain performance could be the results of changes in transcriptomic expression. Additionally, future studies investigating in greater detail the time course of transcriptomic changes will be also necessary.

## 5. Conclusions

In this study, we revealed that anodal tDCS modified the expression of a variety of genes in the cerebral cortex. Our data provides transcriptomic evidence to explain the importance of the current intensity of anodal tDCS on the pattern of gene induction. These results show an increased number of transcriptomic changes that are related to neuronal function and identified specified pathways that could contribute to the beneficial effects of tDCS on brain performance. Thus, noninvasive tDCS can be utilized to modulate gene expression within the brain and the pattern and magnitude of the transcriptomic response to tDCS are dependent upon the current intensity applied.

## Figures and Tables

**Table 1:** Genes in the cell communication subcategory that were detected from RNA-Seq data sets.

Groups	List of genes
250 *μ*A only	Adap2, Blnk, Cd27, Cx3cr1, S1pr4, Igsf1, Lair1, Ltb, N*μ*Ak2, Osmr, P2ry14, Phkg2, Birc3, Ccdc88b, Efcab7, Gem, Il10rb, Prkcd, Lrrc32, Nmi, S100a13, Slamf9, Tnfrsf14, Lck, Sik1, Stat1, Sucnr1, Tssk4

500 *μ*A only	Ibsp, Clec4a2, Fzd6, Gabrg1, Gabrr3, Il10ra, Mc4r, Cd244, Ntf3, P2ry12, Pth2r, B3gnt4, Bmp8a, Dock2, Gsg2, Pcdhb8, Pcdhga1, Pcdhgb7, Unc5cl, Rps6ka6, Myo1b

2000 *μ*A only	Adamts1, Adamtsl4, Angpt2, Angptl2, Anxa1, Asgr1, Bmp5, C5ar2, Calcr, Calca, Cd40, Cxcl11, Cmklr1, Cartpt, Col1a2, Cubn, Cxcl10, Fmod, Frzb, Lgals3bp, Gadd45g, Gdf15, Cxcl1, Igfbp2, Itgb4, Il1b, Prg4, Llgl2, Mdk, Map3k8, Ogn, Pdgfrl, Pcolce, Pomc, Ptger2, Ptger4, Clec4a3, Col8a2, Csf2ra, Cyr61, Gpr31, Map3k19, Msr1, Rab20, S100a11, S100a6, Ssc5d, Selp, Sfrp1, Slc6a20, Slc22a6, Socs1, Cd4, Tnfsf4, Tnf, Fes, Plau, Wisp2

Intersection of 250 *μ*A & 500 *μ*A	Angptl4, Asgr2, Ccr5, Cd48, Cp, Cxcl9, Csf3r, C1qb, Clec4a1, Drd4, Fgl2, Havcr2, Itgb2, Sell, Lcp2, Myo1f, Prss12, Lcn2, Nfkbia, P2ry13, Pla2g2d, Plek, Btg2, Card11, Cfh, Dock8, Dtx3l, Gpr132, Gpr84, Rac2, Rhoh, S100a9, Slamf8, Tlr3, Vav1, Tnfrsf1b, Tacstd2, Fgr, Hck, Lyn

Intersection of 250 *μ*A & 2000 *μ*A	Il2rg, Abi3, Asgr2, Bcl2a1, C5ar1, Cp, Clic1, Csf3r, C1qb, Clec4a1, Tspan8, Cfb, Fgl2, Fbln2, Hcar2, Itgb2, Ifi35, Lcp2, Mgp, Myo1f, P2ry6, Plek, Ptgds, Arhgdib, Btg2, Card11, Cfh, Dock8, Dtx3l, Gpr132, Gpr84, Mlkl, Rac2, Rasal3, Rhoh, Slamf8, Tagap, Tlr3, Vav1, Ripk3, Socs3, Junb, Tgfb1, Tnfrsf1b, Tacstd2, Fgr, Hck, Lyn

Intersection of 500 *μ*A & 2000 *μ*A	Asgr2, Cnr2, Cp, Csf3r, C1qb, Clec4a1, Fgl2, Gpr183, Gna15, Itgb2, Lcp2, Myo1f, Ncf1, Nlrc4, Plek, Btg2, Card11, Cfh, Dock8, Dtx3l, Fyb, Gpr132, Gpr84, Naip6, Pram1, Rac2, Rhoh, Slamf8, Tlr3, Vav1, Tnfrsf1b, Tacstd2, Fgr, Hck, Lyn

Intersection of all current intensities	Asgr2, Cp, Csf3r, C1qb, Clec4a1, Fgl2, Itgb2, Lcp2, Myo1f, Plek, Btg2, Card11, Cfh, Dock8, Dtx3l, Gpr132, Gpr84, Rac2, Rhoh, Slamf8, Tlr3, Tnfrsf1b, Tacstd2, Fgr, Hck, Lyn

**Table 2:** Genes in the primary metabolic process subcategory that were detected from RNA-Seq data sets.

Groups	List of genes
250 *μ*A only	Alox5ap, Nostrin, Rpl3l, Pdlim1, Hsd17b1, Pla2g7, Birc3, Cst7, RGD1564980, RGD1564138, LOC100360154, Tmem176b, Slc27a2, Tgm2, Tinagl1, Prkcd, RGD1563220, N*μ*Ak2, Cbr3, Alx3, Ctss, Cyp2j4, Si, RGD1562399, RGD1565048, Apol3, Ddx43, RGD1565844, Cmbl, Npc2, S100a13, Helz2, Phkg2, Vwf, Batf2, Lypd8, Sik1, Parp12, Plat, Tssk4, Rpl39, Rpl30, Slamf9, Batf, Lck, Apoa5, Cx3cr1, Faxdc2, Rps15al2, Upp1, Cst3, Mov10, Stat1, Trim21, Herc6, LOC100361854, Rpl35a

500 *μ*A only	Mcmdc2, Gsg2, Rad51c, Smyd1, Phox2a, Zfp711, Cnot6l, LOC100361079, Ftcd, Rps6ka6, Cd55, B3gnt4, LOC102550734, Lgsn, LOC100360611, Gen1, Itih4, Zfp846, LOC100912373, Zfp961, Cd244, Napsa, Mgam, Bmp8a, RGD1562923, Lipm

2000 *μ*A only	Wfikkn2, Osr1, Asf1b, Fes, Pcolce, Cubn, Adgb, C3, Foxc2, Cmklr1, Cpz, Plau, Bmp5, Maff, Zmynd15, Uba7, Fmod, Mybl2, Fut4, Aebp1, Enpp3, Ube2c, Ucp2, Srpx, Bhmt2, Cyp1b1, Selp, Nkx2-1, Dct, Tcea3, Klc3, Ecel1, Adamtsl4, Pim1, Adamts1, Pdlim2, Pdgfrl, Mcm3, Ptgr1, Map3k8, Hk3, Ada, Mcm5, Pbk, Ssc5d, Gdf15, Isg15, A2m, Hlx, Fmo3, Map3k19, Slc17a9, Abca4, Anxa1, Slc22a6, Slc5a5, Apol9a, Ogn, Foxd1, Alas2, Lgals3bp, Tbxas1, S100a11, Twist1, Ppp1r3b, S100a6, Igf2, LOC100363469, Cfd, Cd68

250 *μ*A *∩* 500 *μ*A	St14, Ube2l6, F10, Vwa5a, Ciita, Sell, Fli1, Cp, Trim55, Aspg, Galns, Ggta1, Rnase6, RGD1563045, Cfh, Ccr5, Rgs1, Fos, Lyn, Slamf8, Nlrc5, Tspo, Aga, Nfkbiz, Amy1a, LOC100361180, Rps6, Ptprc, Anxa3, Cd48, Fgr, Lcn2, Pla2g2d, Irf8, Rhbdf2, S100a9, Dtx3l, Msh5, Nlrp3, Nfkbia, Hck, Prss12

250 *μ*A *∩* 2000 *μ*A	Oas1a, St14, Anxa2, Ptpn6, Ube2l6, F10, Junb, Arpc1b, Ciita, Cp, Trim55, Aspg, Psme1, Ch25h, Ggta1, Tgfb1, Tmem176a, Clic1, Cfh, Cfb, Oasl, Ptgds, Parp14, Hspb1, Ctsz, Rgs1, LOC297756, Fos, Lyn, Slamf8, Laptm5, C1s, Ctsh, Oasl2, Mlkl, Nlrc5, A3galt2, Ptges, Tspo, Hpgd, Aga, Ifi30, Col6a3, Cd6, Slc7a8, Parp9, Nfkbiz, Ptprc, Anxa3, Apold1, Fgr, Cpxm2, Irf8, Tmprss2, Rhbdf2, Spi1, Ctsc, Ripk3, Dtx3l, Hcls1, Bcl3, Tap1, Nlrp3, Atf3, Parp10, Rasal3, Hck, Oas1k, Cebpd

500 *μ*A *∩* 2000 *μ*A	Hvcn1, St14, Ube2l6, F10, Ciita, Cp, Trim55, Aspg, Ggta1, Cfh, Nlrc4, Rgs1, Fos, Lyn, Slamf8, Nlrc5, Tspo, Aga, Npas4, Nfkbiz, Ptprc, Anxa3, Fgr, Irf8, Rhbdf2, Apobec1, Dtx3l, Nlrp3, Hck, Naip6, Cebpd

250 *μ*A *∩* 500 *μ*A *∩* 2000 *μ*A	St14, Ube2l6, F10, Ciita, Cp, Trim55, Aspg, Ggta1, Cfh, Rgs1, Fos, Lyn, Slamf8, Tspo, Aga, Nfkbiz, Ptprc, Anxa3, Fgr, Irf8, Rhbdf2, Dtx3l, Nlrp3, Hck

**Table 3:** Genes in the receptor subcategory that were detected from RNA-Seq data set.

Subcategory name (accession)	Groups (number of genes)	List of genes
G-protein coupled receptor (PC00021)	250 *μ*A only (4)	Sucnr1, P2ry14, S1pr4, Cx3cr1
	500 *μ*A only (7)	Fzd6, Pcdhb8, Pcdhga1, Mc4r, Pth2r, P2ry12, Pcdhgb7
	2000 *μ*A only (10)	Cmklr1, Calcr, Ptger2, Gprc5a, Sfrp1, Frzb, Ptger4, Mrgprf, Gpr31, C5ar2
	Intersection of 250 *μ*A & 500 *μ*A (5)	Ccr5, P2ry13, Gpr84, Gpr132, Drd4
	Intersection of 250 *μ*A & 2000 *μ*A (6)	C5ar1, P2ry6, Gpr18, Gpr84, Gpr132, Hcar2
	Intersection of 500 *μ*A & 2000 *μ*A (4)	Cnr2, Gpr84, Gpr132, Gpr183
	Intersection of all current intensities (2)	Gpr84, Gpr132

Cytokine receptors (PC00084)	250 *μ*A only (12)	Igsf1, Tapbpl, Lair1, Il10rb, RT1-N3, Tnfrsf14, Il4r, Siglec1, Slamf9, Cx3cr1, Osmr, Cd27
	500 *μ*A only (4)	Il10ra, Clec4a2, Cd244, Cd79b
	2000 *μ*A only (8)	Cmklr1, LOC100364500, Clec4a3, Il21r, Cd4, Cd40, Asgr1, Csf2ra
	Intersection of 250 *μ*A & 500 *μ*A (10)	Csf3r, Clec4a1, Ccr5, Slamf8, Lag3, Csf2rb, Cd48, Havcr2, Tnfrsf1b, Asgr2
	Intersection of 250 *μ*A & 2000 *μ*A (9)	Csf3r, Clec4a1, Cd33, Slamf8, Csf2rb, Tnfrsf1b, Cd22, Asgr2, Il2rg
	Intersection of 500 *μ*A & 2000 *μ*A (6)	Csf3r, Clec4a1, Slamf8, Csf2rb, Tnfrsf1b, Asgr2
	Intersection of all current intensities (6)	Csf3r, Clec4a1, Slamf8, Csf2rb, Tnfrsf1b, Asgr2

Ligand-gated ion channel (PC00141)	500 *μ*A only (2)	Gabrr3, Gabrg1

Protein kinase receptor (PC00194)	2000 *μ*A only (2)	Pim1, Pdgfrl

**Table 4:** GO enrichment analyses of 2 modules that have the top 2 highest associations with tDCS intensity.

Term name	Term ID	Module	Number of module genes in the term	Enrichment *p*	Bonferroni *p*	Rank in the module
Extracellular matrix	GO:0031012	Brown	68	1.86*E* − 21	2.97*E* − 17	1
Proteinaceous extracellular matrix	GO:0005578	Brown	57	5.12*E* − 20	8.19*E* − 16	2
Extracellular space	GO:0005615	Brown	116	8.65*E* − 17	1.39*E* − 12	3
Vasculature development	GO:0001944	Brown	85	1.84*E* − 15	2.95*E* − 11	4
Tissue development	GO:0009888	Brown	178	3.51*E* − 15	5.63*E* − 11	5
Response to lipid	GO:0033993	Brown	119	4.40*E* − 14	7.04*E* − 10	6
Response to organic substance	GO:0010033	Brown	245	8.04*E* − 14	1.29*E* − 09	7
Extracellular region	GO:0005576	Brown	299	8.66*E* − 14	1.39*E* − 09	8
Wound healing	GO:0042060	Brown	60	2.25*E* − 13	3.61*E* − 09	9
Blood vessel development	GO:0001568	Brown	78	2.30*E* − 13	3.68*E* − 09	10
Response to drug	GO:0042493	Dark grey	23	1.68*E* − 07	0.002690054	1
Eosinophil chemotaxis	GO:0048245	Dark grey	5	2.84*E* − 07	0.004554619	2
Eosinophil migration	GO:0072677	Dark grey	5	1.39*E* − 06	0.022334475	3
CCR chemokine receptor binding	GO:0048020	Dark grey	5	3.17*E* − 06	0.050721539	4
Heparin binding	GO:0008201	Dark grey	9	4.37*E* − 06	0.070004182	5
Glycosaminoglycan binding	GO:0005539	Dark grey	10	7.19*E* − 06	0.115057731	6
Response to nicotine	GO:0035094	Dark grey	7	8.37*E* − 06	0.133943241	7
Chemokine activity	GO:0008009	Dark grey	5	1.16*E* − 05	0.186056475	8
Cerebrospinal fluid secretion	GO:0033326	Dark grey	3	1.79*E* − 05	0.286908137	9
CCR1 chemokine receptor binding	GO:0031726	Dark grey	3	1.79*E* − 05	0.286908137	10
